# Assessment of Bacterial Contamination and Antimicrobial Resistance of *Escherichia coli* Isolates from Slovak Dairy Farms

**DOI:** 10.3390/ani14213095

**Published:** 2024-10-26

**Authors:** Nikola Dančová, Gabriela Gregová, Tatiana Szabóová

**Affiliations:** Department of Public Veterinary Medicine and Animal Welfare, The University of Veterinary Medicine and Pharmacy in Košice, 041 81 Košice, Slovakia; nikola.dancova@uvlf.sk (N.D.); gabriela.gregova@uvlf.sk (G.G.)

**Keywords:** antimicrobial resistance, bioaerosol, *E. coli*, feces, cattle

## Abstract

The conditions in livestock housing are suitable for the survival of airborne microorganisms, mainly due to high temperatures, humidity, and the presence of organic material. The total count of airborne bacteria concentrations in cattle farms ranged from 3.01 log_10_ CFU/mL to 6.90 log_10_ CFU/mL; for coliform bacteria, they were from 2.18 log_10_ CFU/mL to 3.34 log_10_ CFU/mL; and for molds, they ranged from 3.00 log_10_ CFU/mL to 4.57 log_10_ CFU/mL. Bacteria resistant to antimicrobial substances and resistance genes can be spread on animal farms. Antimicrobial resistance in ubiquitous *Escherichia coli* isolated from cattle feces was investigated. Minimum inhibitory concentration (MIC) testing was utilized to identify phenotypic resistance profiles, and the PCR method was employed to detect the presence of resistant genes. A higher percentage of resistance was found to amikacin (65%), tetracycline (61%), streptomycin (56%), ampicillin (55%), and nalidixic acid (45%). Multidrug resistance was determined in up to 64.3% of the isolates studied. The most widespread resistance genes were *bla*_TEM_ (85.7%), *sul*2 (66.7%), *tet*B (52.38%), and *sul*1 (47.6%). We found that 4.8% of the *E. coli* isolates had the *bla*_CMY_ gene. We found that, despite phenotypic resistance, *E. coli* isolates do not necessarily carry genes conferring resistance to that particular antimicrobial agent.

## Simple Summary

Intensive livestock farming is now widespread around the world and is also a significant producer of pollutants. Emissions of airborne bacteria or bioaerosols affect not only the health of animals but also affect their products, which can have serious consequences for public health. In our study, we focused on the detection of bacterial pollution on three cattle farms located in Slovakia using a MAS-100 Eco^®^ air sampler. A high total count of bacteria and molds in the air and deficiencies in the ventilation system were detected, which created suitable conditions for their survival. Microbial resistance poses another severe risk to global health. Antimicrobial-resistant bacteria carrying antimicrobial resistance genes can be introduced into the environment through animal feces. Ubiquitous *E. coli* is the most commonly used indicator bacteria for monitoring the spread of antimicrobial resistance and is a frequent carrier of various resistance genes to antimicrobial agents. We phenotypically and genotypically confirmed the resistance of *E. coli* isolated from cattle feces to the antimicrobial agents investigated. Our findings revealed the presence of multidrug-resistant *E. coli* strains.

## 1. Introduction

The dairy industry plays a vital role in the agricultural sector of the Slovak Republic, contributing significantly to the economy and the population’s nutrition [1]. However, intensive animal husbandry releases high concentrations of air pollutants such as bioaerosols into the environment. In agricultural livestock housing, bioaerosols are a complex mixture of organic dust, biologically active components (e.g., endotoxins and mycotoxins), and microorganisms (e.g., bacteria, fungi, and viruses). The emission and transport of bioaerosols are associated with the development of diseases in animals and also negatively affect the health of farmers and residents of the surrounding areas of livestock enterprises [2].

The concentrations and types of microorganisms in indoor livestock housing air are affected by technical factors (i.e., the type and age of a building), the number of inhabitants, the heating and ventilation systems, and microclimatic conditions such as the temperature, relative humidity, gas concentration, lighting, and dust concentration. Improper working practices and unhygienic conditions may be causes of considerable microbial air pollution [3]. In particular, bacteria are very abundant on farms, with the most important reservoirs and vectors being the farm animals and their feces [2]. Pathogenic zoonotic bacteria in the environment of animal farms include *Staphylococcus* spp., *Enterococcus* spp., *E. coli*, *Salmonella* spp., *Campylobacter* spp., *Listeria* spp., *Pseudomonas* spp., and others [4]. Animal feed is often contaminated with molds, especially those of the genera *Aspergillus*, *Penicillium*, *Fusarium*, *Mucor*, or *Cladosporium* [3].

For these reasons, farm animals are given antimicrobial substances for the treatment and prevention of several infectious diseases. In the past, these agents were also given to promote their growth. The overuse and misuse of antimicrobial substances in animal husbandry have significantly contributed to the emergence and spread of antimicrobial resistance in bacteria, such as *E. coli*. The use of antimicrobials in the livestock industry is substantially greater than in human medicine, with an estimated annual consumption of over 60,000 tons in intensive livestock farms worldwide. Furthermore, resistant bacteria can be shed and released into the environment, posing a risk of spreading antimicrobial resistance according to the concept of “One Health” [5].

The majority of *E. coli* strains are non-harmful bacteria that coexist with humans, animals, and birds in their digestive systems. These commensal forms of *E. coli* are widely present on farms and are commonly used to monitor the spread of antimicrobial resistance in various environments and host species. They also frequently carry different antimicrobial resistance genes. Nonetheless, *E. coli* can also be harmful and cause a variety of intestinal or extra-intestinal infections [6,7]. The current presence of multidrug-resistant *E. coli* worldwide is alarming. Until recently, *E. coli* was susceptible to almost all clinically important antimicrobial drugs. However, this bacterium has shown an impressive ability to receive and donate resistance genes, mainly through horizontal gene transfer. The most problematic mechanisms in *E. coli* strains involve gaining genes resistant to β-lactams. Furthermore, *E. coli* originating from animals frequently exhibits resistance to other antimicrobial agents, such as tetracyclines, quinolones, aminoglycosides, phenicols, sulfonamides, trimethoprim, and fosfomycin. Reports of *E. coli* resistance to colistin have also emerged from various parts of the world [8].

Animal feces are commonly utilized as fertilizer to enhance the nutrient content of agricultural soils. However, animals release many microorganisms in their feces, particularly two prevalent zoonotic bacterial species, *E. coli*, and *Salmonella* spp. The application of untreated feces as fertilizer presents a significant risk of environmental contamination, including that of soil, crops, water, and air, with resistant microorganisms, antimicrobial resistance genes, and antimicrobial residues [4]. According to Gou et al. [9], following the administration of antimicrobial substances to farm animals, 75–90% of these substances are excreted into the environment through urine and feces. Antimicrobial resistance in the farm environment and the potential for its further spread are becoming increasingly concerning as part of the “One Health” concept, which connects the health of humans, animals, and the environment. Antimicrobial-resistant *E. coli* poses a threat to animal health, which also raises worries for human health since these resistant strains can disseminate through the food chain or through direct animal contact [10].

The aim of this study was to determine the level of air pollution of bacteria and molds in the livestock environment in order to estimate the health risk of microbiological air pollution. In addition, we aimed to isolate *E. coli* from cattle feces and to perform phenotypic and genotypic analysis of the antimicrobial resistance of these bacteria.

## 2. Materials and Methods

### 2.1. Collection, Processing and Identification

This study was carried out in 2023 on three dairy cattle farms in the eastern part of the Slovak Republic. On average, the farms housed approximately 200 dairy cows in free-range housing in boxes with deep bedding and natural ventilation. Samples of the air and feces were taken from the animal houses. Using a MAS-100 Eco^®^ air sampler (Merck, Darmstadt, Germany), aerosol samples were taken directly into Petri dishes containing the following agars: Meat Peptone Agar (HiMedia, Mumbai, India), Endo Agar (HiMedia, Mumbai, India), and Sabouraud Agar (Oxoid, Basingstoke, UK). The selective media and incubation conditions applied to carry out microbiological analysis are presented in Table 1. The counts of microorganisms were adjusted using the correction tables and recalculated per 1 m^3^ of air. The microclimate’s physical parameters were also measured using a thermo-hygrometer (Testo, Titisee-Neustadt, Germany) to record the ambient temperature and relative humidity throughout the day.

Seventy samples of feces were collected as soon as possible after defecation in sterile plastic specimen boxes and transported to the laboratory under aseptic conditions. After being weighed and diluted 1:10 with distilled water, the samples were homogenized. Following the guidelines of ISO 6887-1 [11], a series of ten-fold dilutions were made from the stock suspension and applied to the surface of Meat Peptone Agar (HiMedia, Mumbai, India), Endo Agar (HiMedia, Mumbai, India), and HiCrome Universal Agar (HiMedia, Mumbai, India). The selected culture media were used to identify *E. coli*. Prepared plates were incubated at 37 °C for 24 h. *E. coli* bacteria on the surface of Endo Agar formed pink to pinkish-red colonies during lactose fermentation, with a typical metallic sheen. On the surface of HiCrome Universal Agar, due to the production of the enzyme ß-galactosidase, which cleaves chromogenic substrate, characteristic purple colonies were formed. On the surface of Meat Peptone Agar, *E. coli* formed large, smooth, white-gray, disk-like mucoid colonies. Enterotest 24 N (Erba Lachema, Brno, Czech Republic) was used to detect and identify the suspected *E. coli* colonies. A single colony of *E. coli* was isolated from each sample.

### 2.2. Antimicrobial Resistance Analysis

The minimum inhibitory concentration (MIC) testing was performed according to Gattringer et al. [12] using the automated diagnostic system Miditech (Bel-Miditech, Bratislava, Slovakia). This diagnostic system consisted of the following antimicrobial substances: ampicillin (AMP), ampicillin + sulbactam (A + IB), ertapenem (ETP), meropenem (MEM), ceftriaxon (CTR), ceftiofur (CFF), ceftazidime + clavulanic acid (CAC), cefquinome (CFQ), gentamicin (GEN), streptomycin (STM), neomycin (NEO), nalidixic acid (NAL), enrofloxacin (ENR), amikacin (AMI), ciprofloxacin (CIP), chloramphenicol (CMP), florfenicol (FLO), tetracycline (TET), co-trimoxazole (COT), and colistin (COL). The results of the MIC values of each antimicrobial substance were interpreted using the EUCAST (14.0 version) [13] clinical breakpoints. MIC xG values represent the geometric mean of antimicrobial MIC values (mg/L) in *E. coli* isolates, as described in our previous study [14].

Genomic DNA of *E. coli* isolates was extracted using the Bacteria DNA Preparation—Solution Kit (Jena Bioscience, Jena, Germany) from overnight cultures grown on Nutrient agar, according to the manufacturer’s instructions. The acquired DNA was analyzed with a NanoDrop One spectrophotometer (Thermo Fisher Scientific, Madison, WI, USA). *E. coli* isolates were subjected to species identification using the PCR method according to Amit-Romach et al. [15]. Antimicrobial resistance genes were detected by polymerase chain reactions (PCRs). The PCR programs began with an initial denaturation step at 94 °C/95 °C lasting 3–15 min, followed by 28 to 35 cycles of DNA denaturation at 94 °C/95 °C for 30–60 s, primer annealing at 54–68 °C (depending on the primers) for 25–60 s, and primer extension at 72 °C for 25 s to 2 min. Following the final cycle, a final extension step at 72 °C for 4–10 min was included. The presence of the following genes was monitored: tetracycline resistance genes—*tet*A and *tet*B; quinolone resistance genes—*qnr*A, *qnr*B, and *qnr*S; genes for resistance to sulfonamides—*sul*1 and *sul*2; β-lactamase-encoding *bla*_TEM_ and *bla*_SHV_; and the ampicillinase gene—*bla*_CMY_. All the primers used for the PCR detection of resistance genes in this study are displayed in Table 2. Detection of amplified PCR products was performed by electrophoresis (Thermo Fisher Scientific, Marietta, OH, USA; Major Science, Saratoga, CA, USA) on 1.5% agarose gels with the addition of GoodView^TM^ fluorescent dye (Amplia s.r.o., Bratislava, Slovak Republic), followed by visualization using a UV transilluminator (Major Science, Saratoga, CA, USA).

## 3. Results and Discussion

### 3.1. Microbial Contaminations and Microclimate Conditions

Intensive animal production is associated with increased production, increased risk of various diseases, and the accumulation of waste, which can negatively affect the environment of the farm itself, including the areas near these farms [22]. The massive overgrowth of airborne microbial communities in livestock production negatively affects animal health, productivity, and welfare, as well as the safety of the food produced [23]. Farmworkers are also exposed to a variety of harmful substances (both organic and inorganic) that lead to respiratory diseases [24].

The microorganism numbers in the air samples and the microclimatic conditions on the cattle farms are presented in Table 3.

Szulc et al. [25] report that an average of 4.4 × 10^4^–1.5 × 10^7^ CFU/m^3^ of microorganisms are found in the air of cattle farm premises. In our study, the concentrations of the total count of bacteria in the cattle farms ranged from 3.01 ± 0.8 log_10_ CFU/mL for calves in milk nutrition period to 6.90 ± 4.1 log_10_ CFU/mL for heifers. We found relatively low concentrations of coliform bacteria (2.18 ± 0.4–3.7 ± 2.7 log_10_ CFU/mL of sample). In the environments with a higher total count of bacteria and molds in the air, deficiencies in the ventilation system were detected, which created suitable conditions for their survival. In general, we found higher numbers of all the studied microorganisms in the air than previously observed by Szulc et al. [25] in cattle on a farm located in central Poland.

In a study by Lange et al. [26], it was found that feed and bedding materials are sources of bioaerosol on dairy farms. The study explored bioaerosol concentrations in a total of 48 dairy cattle barns and demonstrated that the concentrations of different groups of microorganisms varied between the barns by two to three orders of magnitude. They also found that among all the farm management practices, the type of fodder had the strongest correlation with the microorganism concentration. The authors revealed that the feed containing the most moisture, such as bulk feed, has the lowest levels of dust and bacteria in the air. This can be attributed to the fact that aerosols from moist feed settle at a higher rate compared to those from dry feed due to hydration. The type of ventilation was also shown to affect the concentrations of Gram-negative bacteria, dust, and endotoxins in the barns.

In confinement livestock housing, effective ventilation is necessary to supply oxygen, eliminate moisture and odors, avoid heat accumulation, and reduce airborne microorganisms [9].

### 3.2. Antimicrobial Resistance of E. coli Isolates

According to previous research, cattle are an important source of antimicrobial-resistant bacteria [6].

A dominant percentage of resistance was observed to the aminoglycoside AMI (65%). High levels of resistance were also found against TET (61%), STM (56%), and AMP (55%). Resistance to quinolones was also confirmed for NAL (45%), CIP (11%), and ENR (5%). The MIC levels for AMP (MIC > 22 mg/L), STM (MIC > 21 mg/L), and AMI (MIC > 24 mg/L) were higher compared with the EUCAST clinical breakpoint [13] for AMP (MIC > 8 mg/L), STM (MIC > 16 mg/L), and AMI (MIC > 8 mg/L).

Multidrug resistance (MDR), i.e., resistance to three or more antimicrobial agents from different groups, occurred in up to 64.3% of the isolates studied. The prevalence of MDR *E. coli* found in cattle from Romania is also high (68%) compared to that in cattle from France (38%) [6].

A positive result was the 100% sensitivity of the investigated isolates to carbapenems (ertapenem and meropenem) and chloramphenicol. Currently, carbapenem resistance in the *Enterobacterales* family is a growing and ubiquitous threat to global health [27].

**Figure 1:**
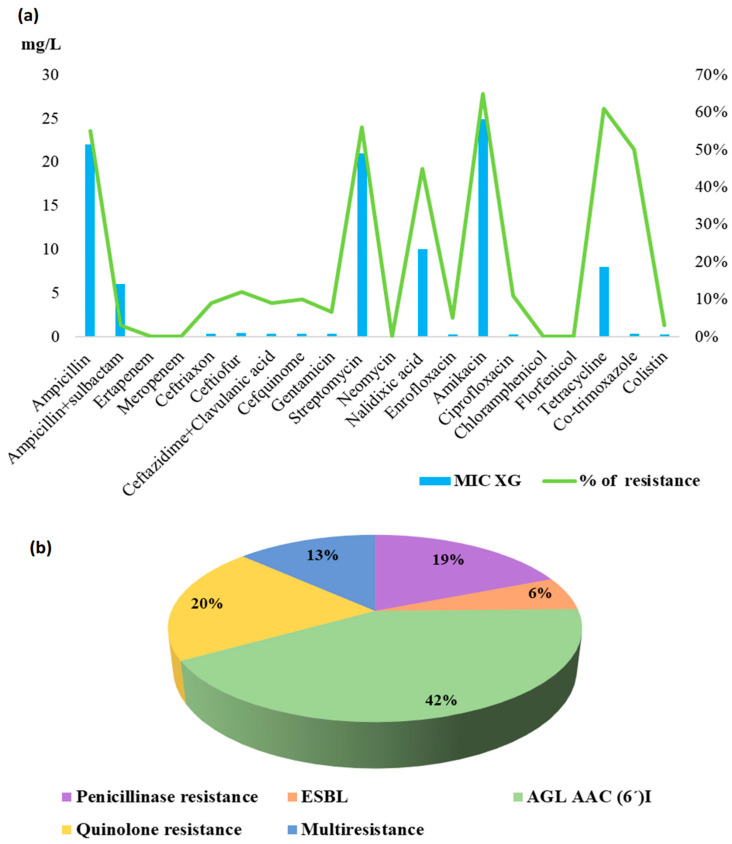
Percentage of resistance and MIC xG values (**a**); mechanism of resistance (**b**) in *E. coli* isolates from cattle feces.

The Miditech system automatically generated the following phenotypic mechanisms of resistance during interpretive reading: AGL AAC (6′)I (42%), i.e., incomplete quinolone cross-resistance also conferring resistance to kanamycin; quinolone resistance (20%); penicillinase resistance (19%); multiresistance (13%); and ESBL, i.e., extended-spectrum β-lactamases (6%). The resistance to the tested antimicrobials with the corresponding MIC xG values and the resistance mechanisms in seventy *E. coli* isolates detected in the cattle feces samples are shown in Figure 1.

The most prevalent infectious disease in dairy animals is mastitis, which has a major negative financial impact on the dairy sector. β-lactams (penicillin, cefapirin, ceftiofur, amoxicillin, hetacillin, and cloxacillin), macrolides (erythromycin), coumarins (novobiocin), and lincosamides (pirlimycin) are among the antimicrobials used to treat it. Infections of the respiratory tract or foot, as well as metritis in dairy cows, are other prevalent infectious diseases in cattle. Ceftiofur and other β-lactams, tylosin, tilmicosin, florfenicol, tetracyclines, and sulfadimethoxine are frequently used to treat respiratory diseases or metritis. Antimicrobial agents such as tetracyclines, lincomycin, β-lactams, and sulfonamides are used to treat foot infections [28].

Twenty-one isolates were selected because they showed resistance to at least one antimicrobial substance, as per the MIC results, or the system automatically generated a probable resistance mechanism for them.

Several different ESBL genes, such as the *bla*_TEM_, *bla*_SHV_, and *bla*_CTX-M_ genes present in bacterial plasmids, encode the ESBL enzyme in *E. coli* [29]. In our study, the most widespread gene was *bla*_TEM_, which we confirmed in 85.7% of the examined isolates; the *bla*_SHV_ gene was detected in more than 23% of the examined isolates. We also detected the presence of the *bla*_CMY_ gene in 4.8% of the *E. coli* isolates. The *bla*_CMY_ *amp*C β-lactamase genes confer broad-spectrum resistance to β-lactam antimicrobial agents, including ceftriaxone and ceftiofur, as well as to β-lactamase inhibitors such as clavulanic acid, posing a potential risk to public health [30]. The *bla*_TEM_ gene was found to be prevalent in *E. coli* obtained from cattle and dairy samples in a livestock farm in Japan. However, none of the samples investigated were positive for the genes *bla*_CTX-M-1_, *bla*_CTX-M-2_, *bla*_CTX-M-8_, and *bla*_CTX-M-9_ [5].

The molecular detection of the *sul*1 and *sul*2 genes resistant to sulfamethoxazole showed a higher prevalence of *sul*2 (66.7%) compared to the *sul*1 gene (47.6%). Similar results were found by Shoaib et al. [31], who identified the *sul*2 gene in 67.3%, *sul*1 in 27.9%, and *sul*3 in 18.1% of tested *E. coli* from samples from a dairy environment.

Resistance to tetracycline is very common in *E. coli* strains isolated from dairy cattle and farm environments, as is the prevalence of genes that confer resistance to tetracyclines [32]. In our study, 14.3% of the *tet*A and 52.4% of the tetracycline-resistant (*tet*B) genes were detected. Our results are also consistent with the findings of Shoaib et al. [31], in which the *tet*B gene was more abundant (70.4%) compared with the *tet*A (11.2%) and *tet*D (0.0%) genes. In addition to *tet*A and *tet*B, Kerluku et al. [7] discovered tetC genes in *E. coli* that were isolated from dairy cows’ feces.

Furthermore, plasmid-mediated quinolone resistance (PMQR) was also observed, as indicated by the presence of *qnr*A (23.8%), *qnr*B (9.5%), and *qnr*S (23.8%) genes in resistant *E. coli* isolates. In the study conducted by Kerluku et al. [7], the *qnr*A gene, responsible for quinolone resistance, was found to have a prevalence of 38.46%. Shoaib et al. [31] discovered a low occurrence of *qnr* genes (*qnr*B 0.34%; *qnr*S 9.43%) in *E. coli* isolates.

As reported by Kerluku et al. [7], the most common resistance genes in *E. coli* from livestock were genes for resistance to ampicillin (*bla*_TEM_, *bla*_SHV_, and *bla*_CMY_), tetracyclines (*tet*A and *tet*B), co-trimoxazole (sulfamethoxazole (*sul*1, *sul*2, and *sul*3) trimethoprim (*dfr*A1 and *dfr*A17)), aminoglycosides (*aph*(3″)-Ia, *aph*(6)-Id, and *aac*(3)-IV), and quinolones (*qnr*A and *aac*(6′)-Ib-cr). *E. coli* strains derived from calves have the primary genes blaCMY, blaCTX-M, *mph*A, *erm*B, *aac*(6′)Ib-cr, and *qnr*S, which confer resistance to AmpC, macrolides, aminoglycosides, and quinolones.

Despite the confirmed phenotypic resistance, not all isolates in our study had the corresponding antimicrobial resistance gene present. The correlation between phenotype and genotype is complex and depends on the methods, antimicrobials, and targets used. When comparing the results of phenotypic and genotypic resistance detection, three common scenarios can occur: (1) the genotype correlates with the phenotype; (2) a resistance gene is detected in an isolate, but phenotypic testing indicates that the isolate is susceptible to the drug; (3) phenotypic testing indicates that the isolate is resistant to the antimicrobial, but the resistance gene is not detected [33]. Several authors believe that a genetic change (mutations or acquisition of genes by horizontal transfer) is required to acquire phenotypic resistance. However, there are also situations where bacteria become temporarily resistant to antimicrobial substances without a genetic change. The most studied are drug indifference, the growth of biofilms, and the phenomenon of persistence. The induction of specific resistance mechanisms, such as chromosomally encoded β-lactamases, can also lead to increased resistance. Additionally, a resistant phenotype can result from a large inoculum that is heterogeneously resistant [34].

**Figure 2:**
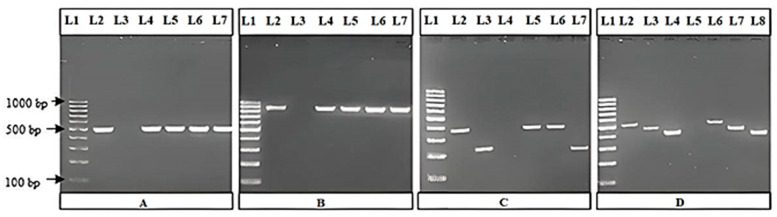
Detection of selected genetic determinants in *E. coli* isolates by PCR.

Table 4 summarizes the antimicrobial resistance profiles of selected *E. coli* isolates. A visualization of the amplified DNA fragments of some of the PCRs performed is shown in Figure 2.

Description:

(A) L1—standard GeneRuler 100 bp DNA ladder, L2—positive control, L3—negative control, L4—L7 isolates positive for *16S rRNA* gene (585 bp);
(B) L1—standard GeneRuler 100 bp DNA ladder, L2—positive control, L3—negative control, L4—L7 isolates positive for *bla*_TEM_ gene (858 bp);
(C) L1—standard GeneRuler 100 bp DNA ladder, L2—positive control (*tet*A), L3—positive control (*tet*B), L4—negative control, L5 and L6—isolates positive for *tet*A gene (372 bp), L7—isolate positive for *tet*B gene (228 bp);
(D) L1—standard GeneRuler 100 bp DNA ladder, L2—positive control (*qnr*A), L3—positive control (*qnr*B), L4—positive control (*qnr*S), L5—negative control, L6—isolate positive for *qnr*A gene (516 bp), L7—isolate positive for *qnr*B gene (469 bp), L8—isolate positive for *qnr*S gene (417 bp).

## 4. Conclusions

The presence of increased concentrations of microorganisms on livestock farms can affect the quality of the environment and use air as a means of spreading. We noticed a high total number of bacteria and molds in the air of diary housing facilities. This was caused by deficiencies in the ventilation system, which created suitable conditions for their survival. Therefore, it is crucial to pay special attention to the air quality as a significant source of pollution for farms and their surroundings.

Despite restrictions in the administration of antimicrobials to livestock, we found the increased resistance of *E. coli* to various antimicrobials, including β-lactams, aminoglycosides, quinolones, and tetracyclines. The application of such untreated cattle manure to soil can introduce resistant *E. coli*, which may contaminate the soil or crops. Also, the improper management or application of cattle manure can lead to the leaching of pathogens into groundwater sources.

It is essential to implement effective farm management practices, such as maintaining hygiene and managing manure, to minimize the risk of transmitting antimicrobial-resistant bacteria to humans. This should be accompanied by the decreased usage of antimicrobials in dairy cows. Furthermore, a comprehensive One Health strategy is required to tackle antimicrobial resistance in humans, animals, and the environment.

## Figures and Tables

**Table 1:** Selective media and incubation conditions used in this study.

Selective Medium	Microorganisms	Incubation	
		Temperature [°C]	Time [h]
Meat Peptone Agar	Total count of bacteria	37	24
Endo Agar	Coliform bacteria	37	24
Sabouraud Agar	Molds	22	72

**Table 2:** Primers used in this study.

Gene	Primer Sequences (5′–3′)	Annealing Temperature (°C)	Product Size (bp)	References
*16S rRNA*	GACCTCGGTTTAGTTCACAGA CACACGCTGACGCTGACCA	55	585	[15]
*tet*A	GGCCTCAATTTCCTGACG AAGCAGGATGTAGCCTGTGC	54	372	[16]
*tet*B	GAGACGCAATCGAATTCGG TTTAGTGGCTATTCTTCCTGCC	54	228	
*qnr*A	ATTTCTCACGCCAGGATTTG GATCGGCAAAGGTTAGGTCA	57	516	[17]
*qnr*B	GATCGTGAAAGCCAGAAAGG ACGATGCCTGGTAGTTGTCC	57	469	
*qnr*S	ACGACATTCGTCAACTGCAA TAAATTGGCACCCTGTAGGC	57	417	
*sul*1	CGGCGTGGGCTACCTGAACG GCCGATCGCGTGAAGTTCCG	55	433	[18]
*sul*2	GCGCTCAAGGCAGATGGCATT GCGTTTGATACCGGCACCCGT	68	293	
*bla*_TEM_	ATGAGTATTCAACATTTCCG CCAATGCTTAATCAGTGAGG	55	858	[19]
*bla*_SHV_	ATGCGTTATATTCGCCTGTG TTAGCGTTGCCAGTGCTC	57	400	[20]
*bla*_CMY_	TGGCCAGAACTGACAGGCAAA TTTCTCCTGAACGTGGCTGGC	64	462	[21]

Description: *E. coli* identification = *16S rRNA*; resistance to tetracycline = *tet*A, *tet*B; quinolone resistance = *qnr*A, *qnr*B, *qnr*S; sulfonamide resistance = *sul*1, *sul*2; β-lactamase-encoding *bla*_TEM_, *bla*_SHV_; and ampicillinase—*bla*_CMY_.

**Table 3:** Average values of concentrations of microorganisms and microclimate parameters in cattle houses.

Place of Sampling	TCB	CB	Molds	T [°C]	RH [%]
	log_10_ CFU/mL				
Delivery room	5.72 ± 2.3	3.34 ± 1.3	4.33 ± 2.1	10.8 ± 1.2	75.4 ± 6.1
Calves in milk nutrition period	3.01 ± 0.8	2.39 ± 1.0	3.00 ± 1.1	7.7 ± 0.8	86.7 ± 10.3
Calves in vegetable nutrition period	4.13 ± 1.7	2.78 ± 0.9	4.16 ± 2.4	8.9 ± 2.7	77.3 ± 5.7
Dairy cows	4.91 ± 1.2	3.15 ± 1.5	4.05 ± 2.8	6.5 ± 3.4	90.5 ± 6.4
Gravid cows	4.21 ± 2.6	2.18 ± 0.4	4.03 ± 1.7	10.1 ± 4.1	69.9 ± 7.2
Heifers	6.90 ± 4.1	3.7 ± 2.7	4.57 ± 2.2	7.4 ± 1.7	88.2 ± 9.8

Values are expressed as average numerical data ± standard deviation. Abbreviations: TCB = total counts of bacteria; CB = coliform bacteria; CFU/g = colony forming units per 1 g of sample; T = temperature; RH = relative humidity.

**Table 4:** Antimicrobial resistance profiles in selected *E. coli* isolates.

No. of Isolate	Phenotypic Resistance	Mechanism of Resistance	Genotypic Resistance
1	AMP, CFT, TZL, NAL, STM, AMI, COT	Multiresistance	*qnr*S, *bla*_TEM_, *sul*1, *sul*2
2	CFT, TZL, STM, AMI, CIP	AGL AAC (6′)I	*qnr*S
3	AMP, CFT, TZL, STM, CIP, TET	Multiresistance; AGL AAC (6′)I	*tet*A, *bla*_TEM_, *qnr*A
4	AMP, GEN, STM, TET, COT	Penicillinase resistance	*tet*B, *bla*_TEM_, *sul*1, *sul*2
5	AMP, CFT, CTR, CFQ	ESBL	without genes
6	AMP, A + IB, CFT, CTR, TZL, CFQ, GEN, STM, CIP, TET, COT	ESBL; multiresistance	*tet*A, *tet*B, *qnr*A, *bla*_TEM_, *bla*_CMY_, *sul*1, *sul*2
7	AMP, CIP, TET	Penicillinase resistance	*tet*B, *qnr*A, *bla*_TEM_, *sul*2
8	AMP, NAL, TET	Quinolone resistance	*tet*A, *qnr*A, *bla*_TEM_, *sul*1, *sul*2
9	AMP, A + IB, CFT, CTR, CFQ, TET, COL, COT	ESBL; quinolone resistance; multiresistance	*bla*_TEM_, *sul*1, *sul*2
10	AMP, A + IB, CFT, CTR, NAL, TET, COL, COT	Multiresistance; quinolone resistance	*bla*_TEM_, *bla*_SHV_, *sul*1, *sul*2

Abbreviations: AMP = ampicillin; A + IB = ampicillin+sulbactam; CFT = ceftiofur; CFQ = cefquinome; CTR = ceftriaxon; TZL = ceftazidime+clavulanic acid; GEN = gentamycin; AMI = amikacin; STM = streptomycin; NAL = nalidixic acid; CIP = ciprofloxacin; TET = tetracycline; COT = co-trimoxazole; COL = colistin.

## Data Availability

Data are contained within the article.
